# Seroprevalence of SARS-CoV-2 anti-nucleocapsid total Ig, anti-RBD IgG antibodies, and infection in Thailand: a cross-sectional survey from October 2022 to January 2023

**DOI:** 10.1038/s41598-023-42754-2

**Published:** 2023-09-20

**Authors:** Jira Chansaenroj, Nungruthai Suntronwong, Sitthichai Kanokudom, Suvichada Assawakosri, Preeyaporn Vichaiwattana, Sirapa Klinfueng, Lakana Wongsrisang, Thanunrat Thongmee, Ratchadawan Aeemjinda, Nongkanok Khanarat, Donchida Srimuan, Thaksaporn Thatsanathorn, Ritthideach Yorsaeng, Apirat Katanyutanon, Wichai Thanasopon, Wichan Bhunyakitikorn, Chaninan Sonthichai, Piyada Angsuwatcharakorn, Withak Withaksabut, Nasamon Wanlapakorn, Natthinee Sudhinaraset, Yong Poovorawan

**Affiliations:** 1 Center of Excellence in Clinical Virology, Department of Pediatrics, Faculty of Medicine, Chulalongkorn University, Bangkok, 10330 Thailand; 2 Center of Excellence in Osteroarthritis and Musculoskeleton, Faculty of Medicine, Chulalongkorn University, King Chulalongkorn Memorial Hospital, Thai Red Cross Society, Bangkok, 10330 Thailand; 3 Chonburi Provincial Public Health Office, Bansuan, Mueang Chonburi, 20000 Chonburi Thailand; 4 Division of Communicable Diseases, Department of Disease Control, Ministry of Public Health, Nonthaburi, Thailand; 5 Vaccine Protection, Division of Communicable Diseases, Department of Disease Control, Ministry of Public Health, Nonthaburi, Thailand; 6 Division of Academic Affairs, Faculty of Medicine, Chulalongkorn University, Bangkok, Thailand; 7 FRS(T), The Royal Society of Thailand, Sanam Sueapa, Dusit, Bangkok, 10300 Thailand

**Keywords:** Infectious diseases, Infectious diseases, Epidemiology, SARS-CoV-2

## Abstract

Seroprevalence studies on SARS-CoV-2 are essential for estimating actual prevalence rates of infection and vaccination in communities. This study evaluated infection rates based on total anti-nucleocapsid immunoglobulin (N) and/or infection history. We determined the seroprevalence of anti-receptor binding domain (RBD) antibodies across age groups. A cross-sectional study was conducted in Chonburi province, Thailand, between October 2022 and January 2023. Participants included newborns to adults aged up to 80 years. All serum samples were tested for anti-N total Ig and anti-RBD IgG. The interviewer-administered questionnaires queried information on infection history and vaccination records. Of 1459 participants enrolled from the Chonburi population, ~ 72.4% were infected. The number of infections was higher in children aged < 5 years, with evidence of SARS-CoV-2 infection decreasing significantly with increasing age. There were no significant differences based on sex or occupation. Overall, ~ 97.4% of participants had an immune response against SARS-CoV-2. The anti-RBD IgG seroprevalence rate was lower in younger vaccinated individuals and was slightly increased to 100% seropositivity at ages > 60 years. Our findings will help predict the exact number of infections and the seroprevalence of SARS-CoV-2 in the Thai population. Furthermore, this information is essential for public health decision-making and the development of vaccination strategies.

## Introduction

Coronavirus disease 2019 (COVID-19) has had devastating consequences worldwide. In January 2023, there were more than 662 million cases and 6.7 million deaths reported worldwide, with more than 4.7 million cases and 33.7 thousand deaths, in Thailand alone^1^. However, the number is likely underestimated by a lack of viral nucleic acid amplification or rapid antigen testing in patients with asymptomatic or mild disease. Serosurveys provide a more accurate estimation of the extent of Severe Acute Respiratory Syndrome Coronavirus 2 (SARS-CoV-2) infections than virological testing and can guide public health decisions^2^.

Serosurveillance is an essential tool for assessing disease burden and helps to determine the transmission pattern of an infectious disease through the community^3–5^. The seroprevalence rate across different countries varies depending on the study population, region, and period^6^. The study of SARS-CoV-2 seroprevalence is critical to monitor the serostatus of SARS-CoV-2 antibodies induced in a population by natural infection, vaccination, or both^7,8^. As previously described, population-based serosurveillance was critical to monitoring the serostatus of individuals during the recent pandemic^9–11^. Anti-receptor binding domain (RBD) antibodies were previously used to identify natural infection and vaccination rates^12^ and are critical to preventing SARS-CoV-2 infection^13^. Anti-nucleocapsid (N) antibodies can be used as a marker to identify previous infection ^14^. Several countries have widely endorsed the use of the inactivated COVID-19 vaccine due to its ability to stimulate the production of SAR-CoV-2-specific anti-RBD. As the inactivated vaccine contains the whole virion, it can induce the production of anti-N similar to those triggered by natural infection. However, previous studies indicated that anti-N induced by inactivated COVID-19 vaccine decline after 3–6 months^15–18^. Conversely, anti-N induced by natural infection remains detectable for more than 6 months, surpassing the duration achieved through vaccination^19–21^.

According to a previous report of SARS-CoV-2 seroprevalence, the actual incidence of SARS-CoV-2 infection is likely much higher than that of the reported cases^2,8^. For example, in the United States, the seroprevalence of antibodies against the SARS-CoV-2 nucleocapsid protein ranged from 3 to 10% in 2020 and reached roughly 20–60% in 2021^22–26^. In Thailand, seropositivity was found to be 1.5–7.5% from May 2020 to May 2021^27^. There has been limited updated information on SARS-CoV-2 seroprevalence. To extend the data from previous serosurveillance studies, we investigated the cross-sectional seroprevalence of SARS-CoV-2 in the representative province, Chonburi, by measuring total anti-nucleocapsid (anti-N) Ig and anti-RBD IgG antibodies against SARS-CoV-2 to estimate infection and seroprevalence in Thailand. This information may help policy makers define vaccination campaigns and implement prevention strategies against COVID-19.

## Results

### Study participants

**Figure 1:**
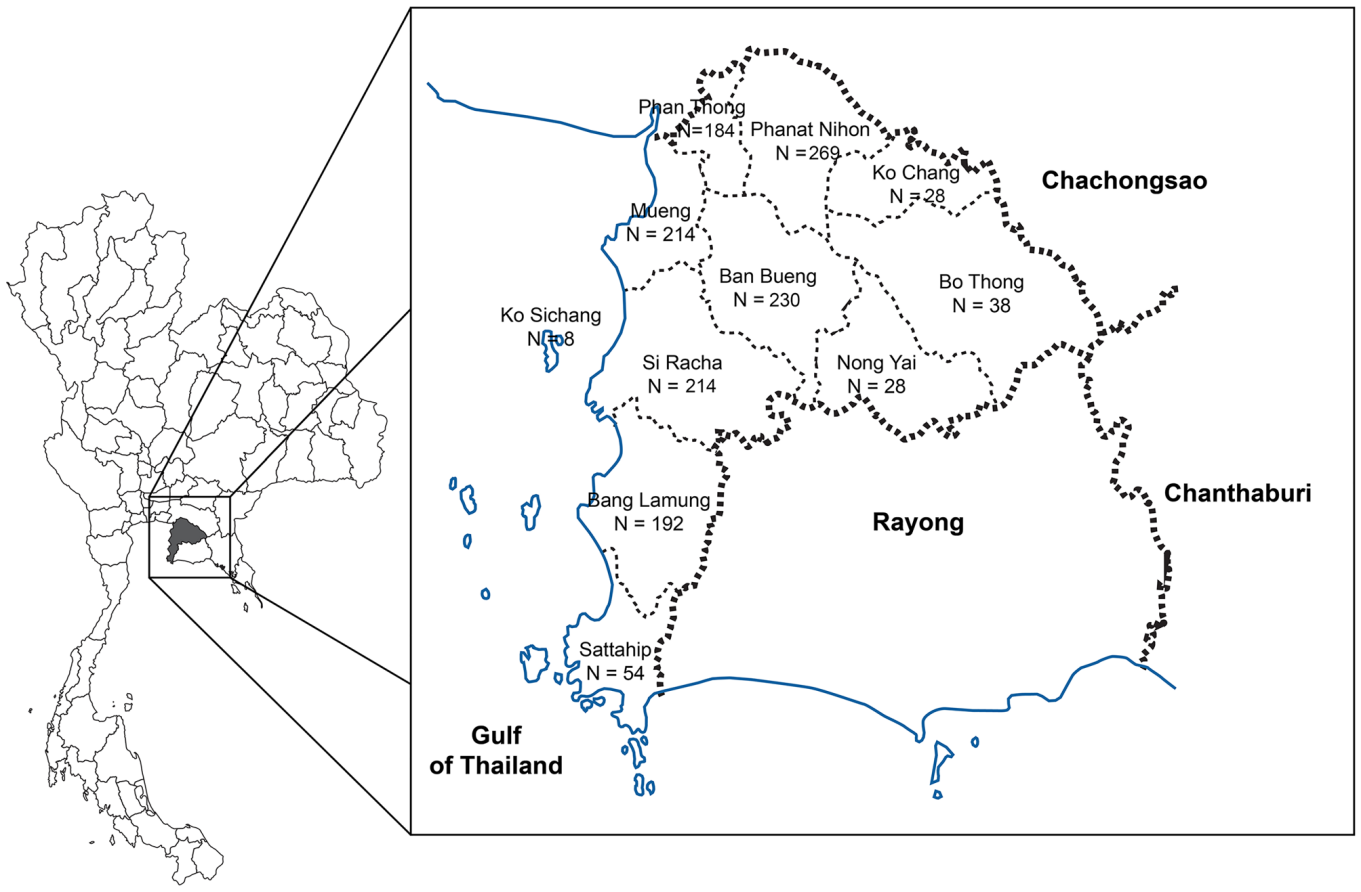
Map of Thailand showing the blood sampling sites in 11 districts, Chonburi province, Thailand. The number of blood samples collected for the individual district is indicated.

A total of 1459 individual serosurveys were evaluated between October 2022 and January 2023. The serosurvey was conducted in 11 districts in Chonburi province and the number of blood samples collected is shown in Fig. 1.

The demographic characteristics of the participants enrolled in this study, including sex, age class, history of infection, and vaccination, are shown in Table 1. The study participants ranged in age from 4 months to 79 years and included 650 men and 809 women. The average age of all participants was 33.0 (standard deviation [SD] = 18.8), and the median was 32.0 (interquartile range: 16 and 48).

**Table 1:** Demographic data of the participants in this study.

Age group, years	Sampling			COVID-19 infection history		COVID-19 vaccination history				
	Total	Male (%)	Female (%)	Not infected (%)	Infected (%)	Not vaccinated (%)	One dose (%)	Two doses (%)	One booster dose (%)	> One booster dose (%)
< 5	96	49 (51%)	47 (49%)	55 (57.3%)	41 (42.7%)	92 (95.8%)	0 (0%)	4 (4.2%)	0 (0%)	0 (0%)
5–10	115	56 (48.7%)	59 (51.3%)	63 (54.8%)	52 (45.2%)	27 (23.5%)	13 (11.3%)	68 (59.1%)	7 (6.1%)	0 (0%)
11–20	218	107 (49.1%)	111 (50.9%)	104 (47.7%)	114 (52.3%)	12 (5.5%)	12 (5.5%)	130 (59.6%)	61 (28%)	3 (1.4%)
21–30	255	103 (40.4%)	152 (59.6%)	99 (38.8%)	156 (61.2%)	5 (2%)	2 (0.8%)	26 (10.2%)	67 (26.3%)	155 (60.8%)
31–40	235	107 (45.5%)	128 (54.5%)	100 (42.6%)	135 (57.4%)	2 (0.9%)	0 (0%)	33 (14%)	66 (28.1%)	134 (57%)
41–50	227	89 (39.2%)	138 (60.8%)	110 (48.5%)	117 (51.5%)	3 (1.3%)	0 (0%)	30 (13.2%)	63 (27.8%)	131 (57.7%)
51–60	204	87 (42.6%)	117 (57.4%)	117 (57.4%)	87 (42.6%)	4 (2%)	4 (2%)	18 (8.8%)	74 (36.3%)	104 (51%)
61–70	92	44 (47.8%)	48 (52.2%)	55 (59.8%)	37 (40.2%)	2 (2.2%)	0 (0%)	21 (22.8%)	40 (43.5%)	29 (31.5%)
> 70	17	8 (47.1%)	9 (52.9%)	11 (64.7%)	6 (35.3%)	1 (5.9%)	0 (0%)	1 (5.9%)	10 (58.8%)	5 (29.4%)
Total	1459	650 (44.6%)	809 (55.4%)	714 (48.9%)	745 (51.1%)	148 (10.1%)	31 (2.1%)	331 (22.7%)	388 (26.6%)	561 (38.5%)

**Figure 2:**
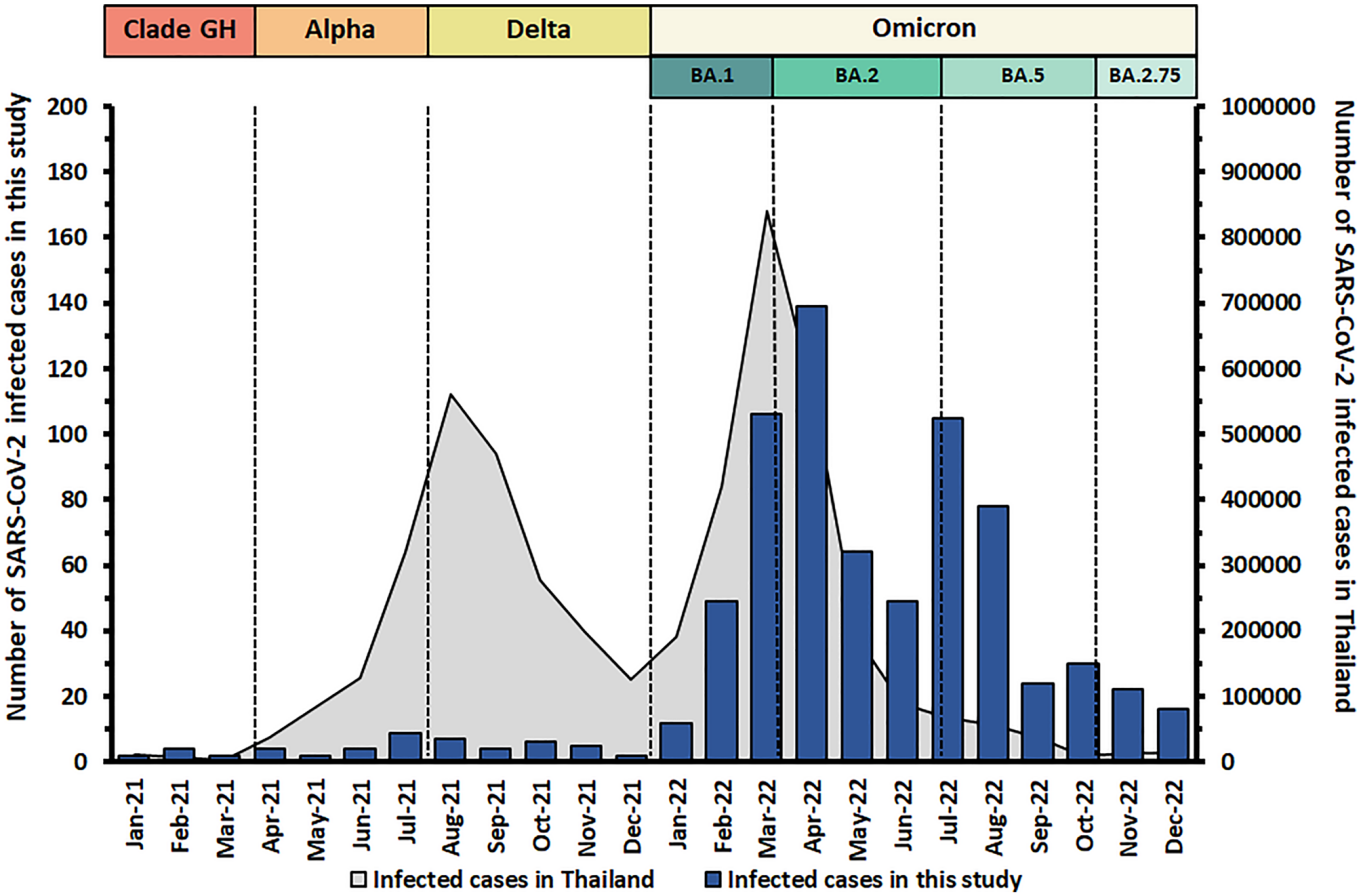
The timeline of infected cases in this study. The left Y-axis represents the number of SARS-CoV-2 infections in this study (bar graph). The right Y-axis represents the number of SARS-CoV-2 infections in Thailand reported by the Ministry of Public Health (area graph). The timeline of infected cases and the duration of the SARS-CoV-2 variant strain outbreaks have been reported in previous studies^28,29^.

Of the 1459 participants, 745 (51.1%) reported a history of a previously diagnosed COVID-19 infection. Most of the participants were infected during the Omicron wave (Fig. 2). A total of 148 (10.1%) participants had not been vaccinated, 31 (2.1%) were vaccinated with only one dose, 331 (22.7%) had received two doses, 388 (26.6%) were vaccinated with one booster dose, and 561 (38.5%) had been vaccinated with more than one booster dose. Of the 1459 individuals included in the analysis, 266 (18.2%; men: 59; women: 207) were healthcare workers and 1193 (81.8%; men: 591; women: 602) were members of the general population.

### The prevalence of infections

**Figure 3:**
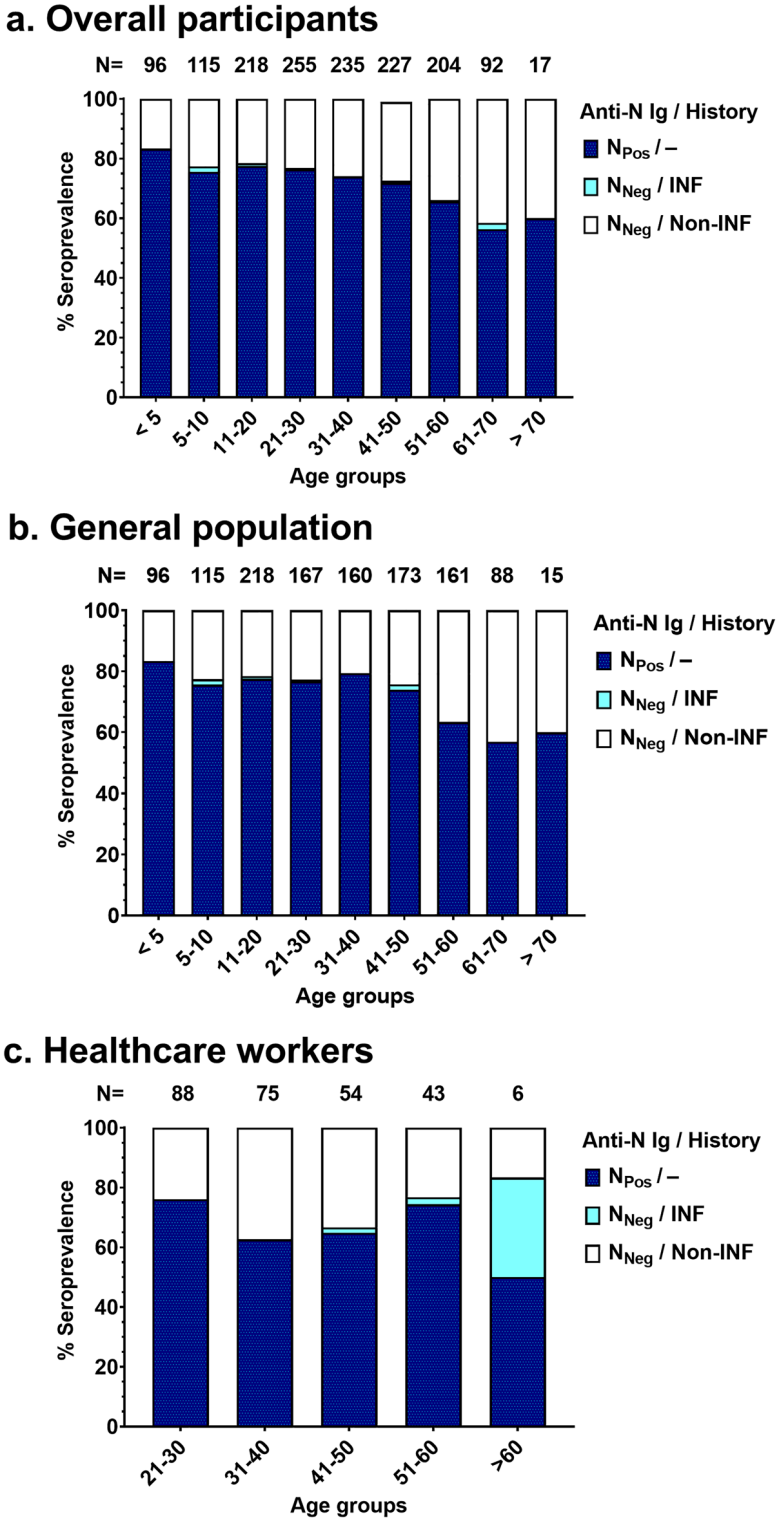
The number of infections measured by anti-N total Ig and/or history of infection (**a**) in all participants, (**b**) in the general population, and (**c**) in the healthcare worker group.

The number of SARS-CoV-2 infections was estimated based on seropositivity of total anti-N Ig antibodies and/or self-reported history of previous SARS-CoV-2 infection. The number of infections stratified by age group is shown in Fig. 3. The total number of total anti-N Ig seropositive and/or COVID-19 infection histories was 73.7% (1076/1459); 74.4% (888/1193) in the general population; and 70.7% (188/266) among healthcare workers. Sex and occupation did not have a significant impact in the risk of developing SARS-CoV-2 infection (*p*-value = 0.552, and 0.128, respectively). The number of infections in each age group ranged from 60.0 to 83.3% and was significantly different between age groups (*p*-value < 0.001). In general, the highest percentage of infections was found in the young age group (83.3%, in children aged < 5 years) and decreased slightly in older age groups (60.0%, in individuals aged > 70 years) (Fig. 3a.). The trend in the number of infections observed in the general population (Fig. 3b) was comparable to that observed among healthcare workers (Fig. 3c). Notably, healthcare workers aged above 60 years old exhibited a significant proportion of individuals with natural infection but anti-N total Ig negative, despite the small sample size (n = 6) (Fig. 3c).

### Seroprevalence of anti-RBD IgG antibodies

**Figure 4:**
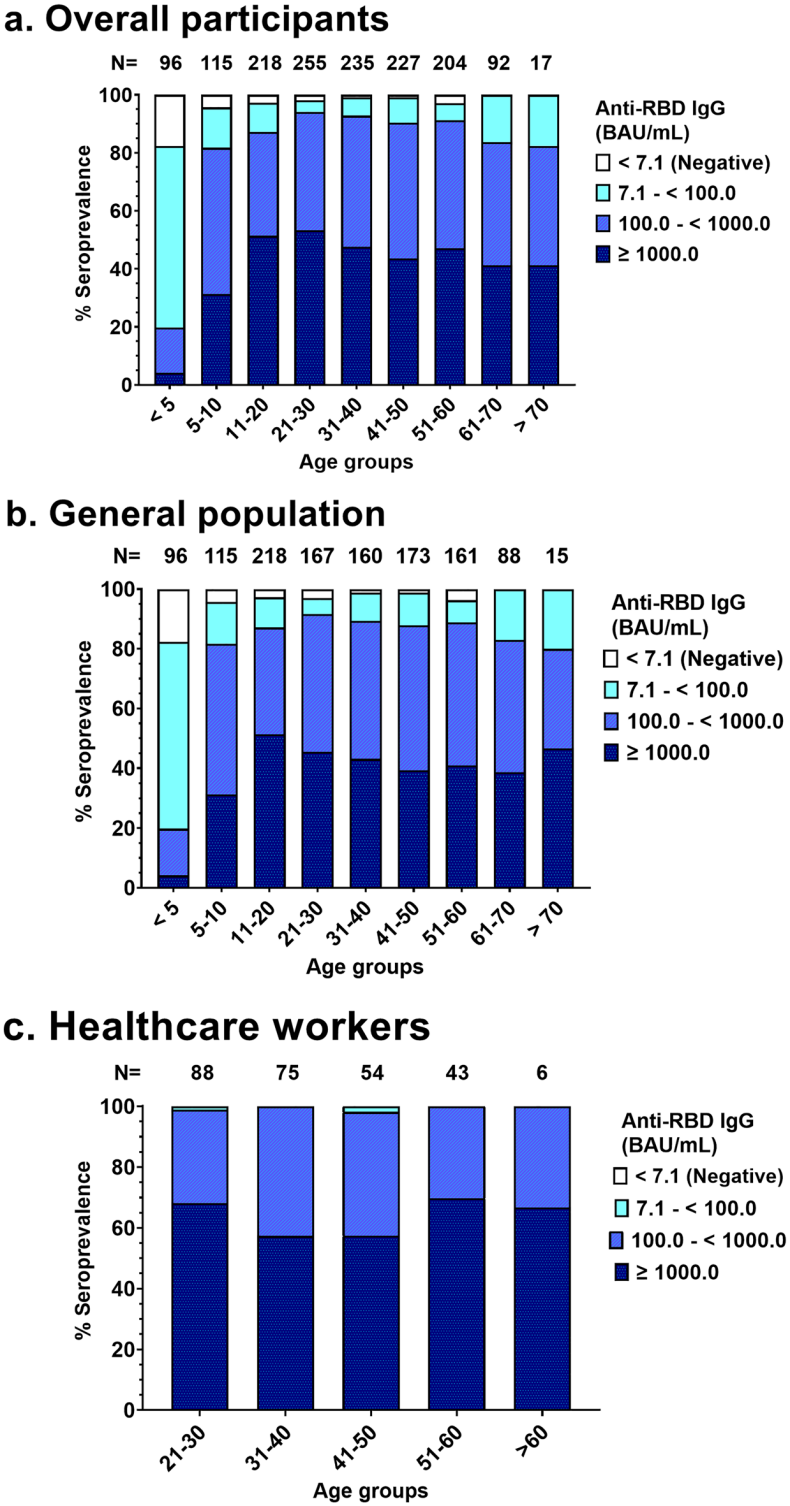
The overall seroprevalence measured by anti-RBD IgG in (**a**) all participants, (**b**) in the general population, and (**c**) among healthcare workers. Anti-RBD concentrations were classified into four ranges: < 7.1 BAU/mL, seronegative; 7.1– < 100 BAU/mL, low titer, 100– < 1000; BAU/mL medium titer, and ≥ 1000 BAU/mL, high titer.

To assess anti-RBD IgG seroprevalence, anti-RBD IgG was measured. The seroprevalence classified by anti-RBD IgG titer for each age group is shown in Fig. 4. Overall, 97.1% (1416/1459) of the participants were anti-RBD IgG seropositive. Of whom, there were 96.4% (1150/1193) of the general population and 100% (266/266) of healthcare workers seropositive. This result found that the relative risk of seropositivity was significantly higher in healthcare workers than in the general population (Relative risk [RR] = 1.23; 95% CI 1.20–1.26, *p*-value < 0.01). Seroprevalence was significantly different in each age group (*p*-value < 0.001) and ranged from 82.3 to 100%. The lowest age stratified seropositivity was observed in individuals under 5 years of age, and it increased slightly to 100% in individuals over 60 years of age. As classified by anti-RBD titer, 43.9% (640/1459) had a high anti-RBD IgG titer, 41.3% (603/1459) had a medium anti-RBD IgG titer, and 11.9% (173/1459) had a low anti-RBD IgG titer. The highest percentage of high anti-RBD IgG titers was found in participants aged 21–30 years (53.3%, 136/255). The anti-RBD IgG titer showed significant differences between the general population and the healthcare worker group (Chi-square = 73.19, *p*-value < 0.001). Many healthcare workers (63.2%, 168/266) showed a high level anti-RBD IgG titer, while most in the general population (42.5%, 507/1193) had a medium level anti-RBD IgG titer.

### Estimated numbers of infections and overall seroprevalence in the Chonburi population

In this study, the number of infections (anti-N positive and/or self-reported history of previous SARS-CoV-2 infection) and anti-RBD seroprevalence were used to predict the number of infections and the overall seroprevalence in the Chonburi population. The percentage positivity rate in each age group was calculated based on the actual numbers reported for the Thai population in official government records for each age group. At the end of 2022, the actual census count in Chonburi province and Thailand reported 1,594,758, and 64,867,406 individuals, respectively. The population was classified according to age group in Table 2. Based on the calculations for each age group, the Chonburi population had approximately 1,154,984 (72.4%) infections and 1,553,969 (97.4%) immune responses to COVID-19. Compared to Thailand, the Thai population reported approximately 46,438,034 (71.6%) infections and 63,333,347 (97.6%) immune responses to COVID-19 (Table 2).

**Table 2:** The approximate number of infections and immune responses for COVID-19 stratified by age in Chonburi and Thailand populations.

Age group, years	Chonburi province					Thailand				
	Population^§^	% Number of infections (our study)	Number of SARS-CoV-2 infections (predict)	% anti-RBD IgG Seropositivity (our study)	Number of positive immune responses for COVID-19 (predict)	Population^§^	% Number of infections (our study)	Number of SARS-CoV-2 infections (predict)	% anti-RBD IgG Seropositivity (our study)	Number of positive immune responses for COVID-19 (predict)
< 5	80,692	83.3	67,216	82.3	66,410	2,767,309	83.3	2,305,168	82.3	2,277,495
5–10	125,258	77.4	96,950	95.7	119,872	4,288,575	77.4	3,319,357	95.7	4,104,166
11–20	199,201	78.4	156,174	97.2	193,623	7,876,321	78.4	6,175,036	97.2	7,655,784
21–30	229,333	76.9	176,357	98.0	224,746	9,092,780	76.9	6,992,348	98.0	8,910,924
31–40	251,349	74	185,998	99.1	249,087	9,311,510	74	6,890,517	99.1	9,227,706
41–50	267,013	73.6	196,522	99.1	264,610	10,146,828	73.6	7,468,065	99.1	10,055,507
51–60	216,941	66.2	143,615	97.1	210,650	9,735,110	66.2	6,444,643	97.1	9,452,792
61–70	130,895	58.7	76,835	100.0	130,895	6,696,235	58.7	3,930,690	100.0	6,696,235
> 70	94,076	58.8	55,317	100.0	94,076	4,952,738	58.8	2,912,210	100.0	4,952,738
Total	1,594,758	72.4	1,154,984	97.4	1,553,969	64,867,406	71.6	46,438,034	97.6	63,333,347

^§^Based on the actual census count in December 2022. Data available from https://stat.bora.dopa.go.th/new_stat^30^.

## Discussion

Serosurveys play an important role in determining the prevalence and monitoring transmission trends of infectious diseases and their burden in the general population or in selected groups^31^. The reported morbidities and mortality of SARS-CoV-2 vary worldwide. As of January 2023, the cumulative incidence reported of SARS-CoV-2 infection cases in Thailand and Chonburi Province was 4.7 million and 23.7 thousand cases, respectively^32^. This population-based cross-sectional serosurvey of SARS-CoV-2 was conducted between 1 October 2022 and 31 January 2023. Anti-N total Ig and anti-RBD IgG were used to detect the number of SARS-CoV-2 infections and anti-RBD seroprevalence in the community population in 11 districts of Chonburi province. According to the findings of this study, Thailand and Chonburi Province had estimated 71.6% and 72.4% of SARS-CoV-2 infections, and 97.6% and 97.4% of their residents had developed an immune response against SARS-CoV-2, respectively. These data strongly suggested that the number of infections reported by the Ministry of Public Health (MoPH) is underestimated.

A previous seroprevalence study performed in Thailand using data from the first three waves of the epidemic between May 2020 and May 2021, found seropositivity of 1.9, 1.5, and 7.5%, respectively^27^. As an extension of that report, this study included data from October 2022 to January 2023—which corresponded to the Omicron wave—and found that approximately 73.7% of the population had experienced SARS-CoV-2 infection. It is possible that almost the entire Thai population was infected with SARS-CoV-2 during the Omicron wave, especially between March and August 2022, as shown in Fig. 2. This is similar to the findings of other studies, which found that antibodies attributed to natural SARS-CoV-2 infection had a detectable high seropositive rate during the Omicron wave^23,33–35^. This positivity rate did not show any significant differences by sex or occupation. The number of infections in the young age group (age < 5 years old) was significantly higher than that in older age groups. These results may be associated with a later implementation of the COVID-19 vaccine in children, especially in those younger than 5 years (as shown in Table 1) and difficulties of controlling social distancing in child communities such as schools. In a small group of healthcare workers aged above 60 years old (n = 6), there was a notable proportion of individuals with natural infection. However, it was observed that anti-N total Ig in some individuals had disappeared, likely due to the waning of anti-N total Ig over time^18^. A larger sample size of participants in this age group will be necessary to understand the dynamics of anti-N total Ig following natural infection and/or vaccination.

At the end of 2021, most of the world’s population may have developed antibodies against SARS-CoV-2, either through infection, vaccination, or both^7,8^. For Thailand and Chonburi province, the full doses of vaccine coverage is 77.2% and 84.9%, respectively, and the respective first booster dose coverage is 48.3% and 58.0%^35^. The seroprevalence of anti-RBD IgG in this study found that 97.1% of the participants had developed antibodies against SARS-CoV-2, consisting of 96.4% of the general population and 100% among healthcare workers. With ongoing efforts for vaccination against COVID-19 and pandemic waves, current levels of hybrid immunity are likely to be substantially higher. This estimated seroprevalence showed a high prevalence rate, similar to previous studies in other countries that reported a seroprevalence rate greater than 90%, such as Chile (98.7% in 2022)^36^, Kenya (90.2% in January–February 2022)^37^, Portugal (95.8% in April–June 2022)^38^, and Switzerland (93.8% in April–June 2022)^38^. Furthermore, the anti-RBD IgG titer showed that almost all participants in the vaccinated age group were seropositive and maintained a medium to high titer, especially among healthcare workers. Thus, the vaccine had the potential to induce an immune response. Almost all healthcare workers may have had the opportunity to receive booster doses of the COVID-19 vaccine, while the general population may face restricted accessibility or hesitancy towards opting for the booster vaccination. According to a prior survey conducted in Bangkok in 2021, 82.2% of the participants expressed agreement with the idea of administering a third vaccine dose to Thai people to combat SARS-CoV-2 with mutations. Meanwhile, 12.2% remained unsure, and 5.6% disagreed with the statement^39^.

Limitations of this study were due to Thailand’s vaccination policy during the first epidemic wave, and the inactivated vaccine being launched for a selected group of individuals at the end of February 2021. As of February 2022, 71.6% of the population received full doses of the vaccination, and the targeting of children aged 5–15 had begun^27^. In March 2022, booster doses of the mRNA vaccine were recommended. In the current study, the participants' vaccination history was obtained solely through questionnaires, which could potentially lead to missing the actual vaccination doses and dates. Since none of the participants in this study reported to have received an inactivated COVID-19 vaccine within six months prior to enrolment, it's possible that all instances of total anti-N Ig positivity were acquired naturally through infection. Although these results cannot fully ensure the representation of the general population in actual numbers, they can significantly affect the comparison of the number of infections and seroprevalence over time. By exploring additional regions in Thailand, the accuracy of seroprevalence data could be improved, leading to a more comprehensive representation of the entire country.

Most participants included in this study achieved a strong immune response against SARS-CoV-2 and had obtained hybrid immunity. This study demonstrates the importance of conducting updated serosurveys. The number of accumulative infections substantially exceeded previous estimates, and overall immunological exposure generated substantial population-level protective immunity. These findings can contribute to guide the planning of public health prevention protocols and the re-evaluation of vaccination strategies in the future, particularly for unvaccinated children.

## Methods

### Study design and ethical considerations

A population-based, age-stratified, cross-sectional investigation was conducted in a selected province using random sampling to estimate the overall burden of the circulation of SARS-CoV-2 infection in Thailand. The study was part of a national serosurvey for vaccine-preventable diseases. The protocol was reviewed and approved by the Institutional Review Board of the Faculty of Medicine of Chulalongkorn University (IRB numbers 0706/65) and was conducted in accordance with the Declaration of Helsinki and the principles of good clinical practice. All participants or their parents were informed of the objectives of the study and written consent was obtained before enrollment in this study.

Chonburi province, located in the eastern Gulf of Thailand approximately 90 km from Bangkok, was chosen as the representative city of Thailand for several reasons. First, its geographical location offers a mix of urban areas and tourist attractions, with Pattaya City serving as a major tourist attraction for beaches, nightlife, shopping centers and entertainment venues. Second, Chonburi province has several growing industrial estates, including the Eastern Seaboard Industrial Estate, while also preserving rural agricultural landscapes where farmers cultivate a variety of crops. Third, the diverse population of the province engages in various occupations, including tourism, industrial manufacturing, and agriculture. This multifaceted agricultural community not only cultivates rice, fruits, and vegetables, but also participates in fisheries, reflecting the Chonburi province's diverse economy. The National Economic and Social Development Council has taken over the Human Achievement Index (HAI) at position 14 in 2019 and the Gross Provincial Product at Current Market Prices (GPP) in the fourth position in 2020^29^. This province had an estimated 1.59 million inhabitants in 2022, residing in 11 districts^40^. Since the COVID-19 pandemic, this province has become a relatively high-risk area, with 23.7 thousand confirmed cases of COVID-19 and 1360 deaths as of 20 January 2023. The total coverage of the COVID-19 vaccine is 84.9%^35^.

### Participants and sample collection

All individuals were included, regardless of age, sex, occupation, history of COVID-19 infection, and COVID-19 vaccination. The inclusion criteria were age between the ages of newborn and 80 years; no immunological disorders such as immunocompetent, malignancy, or severe hematologic disorder; and no history of other chronic diseases. Those who had congenital defects, chronic diseases, or any disease posed a risk of venipuncture or other blood disorders were excluded. A questionnaire was administered. COVID-19 vaccination information and infection history were collected to characterize the demographic and clinical information about the participants.

Two groups of participants, consisting of the general population and healthcare worker groups, were included. The study population was stratified by age, and the cluster random sampling method was used to enroll participants. The stratified clusters within the 11 districts were divided into urban and rural strata. From each district, each cluster was selected using the probability-proportionate-to-size sampling method. Healthcare workers were selected from distinct-level health facilities within each district using consecutive sampling in each district.

Blood samples (3–5 mL) were collected after participants gave their informed consent and fully responded to the questionnaire. Blood samples were centrifuged to collect serum samples, aliquoted and in stored at − 20 C until laboratory testing.

### Serological assays

#### Antinucleocapsid testing and/or a history of infection to assess the number of infections

Anti-N seropositive was investigated using the electrochemiluminescence immunoassay (ECLIA) (Elecsys® Anti-SARS-CoV-2 N, Roche Diagnostics GmbH, Mannheim, Germany) to detect anti-N total Ig against the original Wuhan strain on the Roche Cobas e411 platform. The results of anti-N total Ig antibodies were evaluated using the manufacturer’s initial cut-off index (COI) of 1.0. The number of infections was estimated based on the number of individuals who tested positive for total anti-N Ig and/or had a history of COVID-19 infection.

#### Anti-RBD testing to evaluate hybrid seroprevalence

To determine SARS-CoV-2 antibodies induced by infection, vaccination and hybrid immunity, all sera samples were quantitatively measured for anti-RBD antibodies against the original Wuhan strain using a chemiluminescence microplate immunoassay (CMIA) (Abbott Architect SARS-CoV-2 IgG-II Quant assay, Abbott Diagnostics, Sligo, Ireland) according to the manufacturer’s instructions. The results are reported as Arbitrary Unit per milliliter (AU/mL). According to the WHO International Standards, AU/mL can be converted to units of binding antibodies per milliliter (BAU/mL) by multiplying by 0.142. The value with ≥ 7.1 BAU/mL was considered positive. The level of anti-RBD IgG is associated with the ability to protect the immune system against COVID-19 infection; therefore, anti-RBD concentrations were classified into four ranges: < 7.1 BAU/mL, seronegative; 7.1– < 100 BAU/mL, low titer; 100– < 1000; BAU/mL, medium titer; and ≥ 1000 BAU/mL, high titer.

### Statistical analysis

Data on the prevalence of SARS-CoV-2 antibodies are presented as numbers and percentages. The number of infections was estimated based on individuals who tested anti-N total Ig seropositive and/or provided a history of infection. The seroprevalence of SARS-CoV-2 antibodies induced by infection, vaccination, and hybrid immunity was estimated based on seropositive anti-RBD IgG. Comparison of the number of infections and anti-RBD IgG seroprevalence between sex and groups of the population was performed using Person’s chi-square test. The relative risk ratio with a 95% confidence interval (CI) was calculated. All statistical analyses were performed with SPSS v23.0 (IBM Corp., Chicago, IL). Figures were generated using GraphPad Prism v9.4.1 (GraphPad Software, San Diego, CA). A *p*-value < 0.05 was considered statistically significant.

## Ethics statement

The study protocol was approved by the Institutional Review Board (IRB), Faculty of Medicine, Chulalongkorn University (IRB number 0706/65).

## Informed consent statement

Informed consent was obtained before participant enrollment. The study was conducted according to the Declaration of Helsinki and the Good Clinical Practice Guidelines (ICH-GCP) principles.

## Data Availability

The authors confirm that the data supporting the findings of this study are available within the article.
